# New Work on Etherization in Child Birth

**Published:** 1848-10

**Authors:** 


*New Work on Etherization in Child birth.*—The Boston Medical and Surgical Journal states that a work on the above subject, illustrated by five hundred and thirty cases, by Walter Channing, M. D., is passing through the press, and will soon be published.

